# Does the Laser-Microtextured Short Implant Collar Design Reduce Marginal Bone Loss in Comparison with a Machined Collar?

**DOI:** 10.1155/2016/9695389

**Published:** 2016-08-31

**Authors:** B. Alper Gultekin, Ali Sirali, Pinar Gultekin, Serdar Yalcin, Eitan Mijiritsky

**Affiliations:** ^1^Department of Oral Implantology, Istanbul University Faculty of Dentistry, Istanbul, Turkey; ^2^Department of Periodontology, Bezmialem Vakif University Faculty of Dentistry, Istanbul, Turkey; ^3^Department of Prosthodontics, Istanbul University Faculty of Dentistry, Istanbul, Turkey; ^4^Department of Oral Rehabilitation, The Maurice and Gabriela Goldschleger School of Dental Medicine, Tel Aviv University, Tel Aviv-Yafo, Israel

## Abstract

*Purpose*. To compare marginal bone loss between subgingivally placed short-collar implants with machined collars and those with machined and laser-microtextured collars.* Materials and Methods*. The investigators used a retrospective study design and included patients who needed missing posterior teeth replaced with implants. Short-collar implants with identical geometries were divided into two groups: an M group, machined collar; and an L group, machined and laser-microtextured collar. Implants were evaluated according to marginal bone loss, implant success, and probing depth (PD) at 3 years of follow-up.* Results*. Sixty-two patients received 103 implants (56 in the M group and 47 in the L group). The cumulative survival rate was 100%. All implants showed clinically acceptable marginal bone loss, although bone resorption was lower in the L group (0.49 mm) than in the M group (1.38 mm) at 3 years (*p* < 0.01). A significantly shallower PD was found for the implants in the L group during follow-up (*p* < 0.01).* Conclusions*. Our results suggest predictable outcomes with regard to bone loss for both groups; however, bone resorption was less in the L group than in the M group before and after loading. The laser-microtextured collar implant may provide a shallower PD than the machined collar implant.

## 1. Introduction

Currently, dental implants are the first choice of treatment for missing teeth [1], and satisfactory osseointegration with minimal crestal bone loss is imperative for long-term success [2, 3]. Machined collar implants have been employed as a traditional collar design for many years and are widely used in implant-supported treatments [1, 2].

Marginal bone loss may occur depending on the implant collar design and the distance between the implant-abutment junction (microgap) and the crestal bone in two-piece implant systems [3]. Supragingival placement of two-piece implants with long machined collars may help decrease the influence of these factors on marginal bone loss [4]; however, such placement is not considered esthetically acceptable. The short-collar machined surface of the implant is highly biocompatible with soft tissue and can be leveled supracrestally and subgingivally to prevent esthetic complications [4]. The attachment between the implant collar and connective tissue acts as a physical barrier and prevents apical migration of epithelial cells and fibroblasts that may cause marginal bone loss [5]. However, the attachment between the machined surface and fibroblasts is weak due to the circular oriented connective tissue fibers around the implant collar, and many factors, such as microgap-oriented peri-implant inflammation, micromovement between the abutment and implant interface, repetitive mastication forces, and daily hygiene applications, may break down this fragile attachment [2, 3, 5].

Novel surface-conditioning treatments for implant collars have been investigated to promote soft and hard tissue attachment around the implant neck to minimize crestal bone loss. Recent studies have demonstrated that laser-microtextured surfaces have physical tissue attachments with connective tissue fibers that are perpendicularly oriented to the implant surface [5]. Laser-microtextured collars may help improve the attachment of bone and connective tissue [6]. Short-collar implants that have strong physical barrier attachments to destructive intrinsic and extrinsic factors may show benefits related to limited marginal bone loss.

The aim of this study was to compare changes in marginal bone loss and the success rate using two different types of short-collar implant (machined versus machined + laser microtextured) subjected to conventional loading at a posterior site. The null hypothesis was that there is no difference in crestal bone resorption between implant collars with different surface-conditioning treatments.

## 2. Materials and Methods

### 2.1. Study Design and Sample Selection

To address the research aims, the authors designed and implemented a retrospective study. The study sample was derived from a population of patients who were required to undergo replacement of missing posterior teeth with implants from December 2010 through December 2011 at the Istanbul University Faculty of Dentistry Department of Oral Implantology. Retrospective chart review data was used for sample collection. The inclusion criteria for the study were as follows: treatment with either one of two types of short-collar implant (i.e., Tapered Internal TLX Laser-Lok or TRX, Biohorizons, Birmingham, AL, USA) in the posterior (premolar and molar) region in either the maxilla or the mandible, age ≥ 18 years, good general health, adequate natural bone for insertion of implants measuring at least 3.8 mm in diameter and 10.5 mm in length (nongrafted sites), requirement of a fixed implant-supported prosthesis in the maxilla or mandible, history of follow-up examinations for at least 3 years after implant placement, and the availability of retrospective chart review data for clinical and radiographic variables during follow-up. The exclusion criteria were as follows: lack of medical records related to the study variables, systemic medical conditions such as diabetes and any medication that may influence soft and hard tissue healing mechanisms, uncontrolled oral hygiene, chronic periodontitis in the remaining teeth, smoking history (≥10 per day), previous surgery at the implant site, and lack of primary stability. The presence of these conditions was ascertained according to retrospective chart data collection. A retrospective chart review of data, with consideration of the inclusion and exclusion criteria, yielded the final sample of eligible patients. The study was conducted in accordance with the Declaration of Helsinki and approved by the Ethics Committee of Bezmialem Vakif University (approved protocol number 2015/71306642-050). Written informed consent was acquired from all patients.

### 2.2. Implant Designs

**Figure 1:**
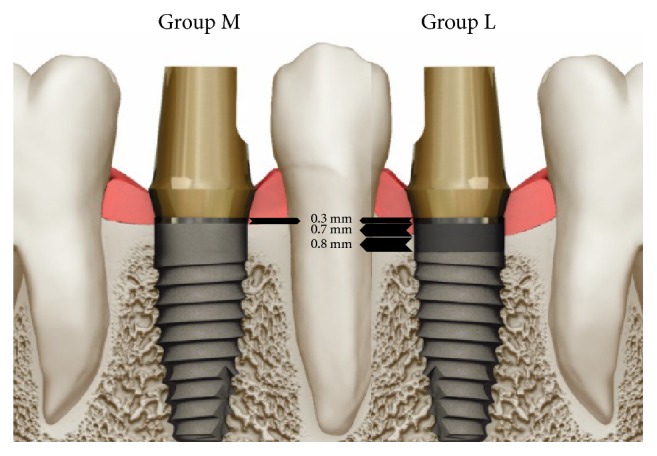
Images of the M group and L group implants used in the study. The M group implants were placed 0.3 mm supracrestally, while the L group implants were placed 1 mm supracrestally.

The short-collared implants in both groups were identical in macrodesign (screw shape), type of abutment connection, and surface characteristics (resorbable blast texturing with a roughness of 0.72–1.34 *μ*m) but had implant collars of different designs; one set had a machined collar (M group: 0.3 mm section of a machined, smooth surface for epithelial tissue attachment [TRX]) and the other had a machined and laser-microtextured collar (L group: 0.3 mm section of a machined, smooth surface; a 0.7 mm section with 8 *μ*m microgrooves for connective tissue attachment; and a 0.8 mm section with 12 *μ*m microgrooves for bone attachment [TLX], as shown in Figure 1.

### 2.3. Surgical Procedure

**Figure 2:**
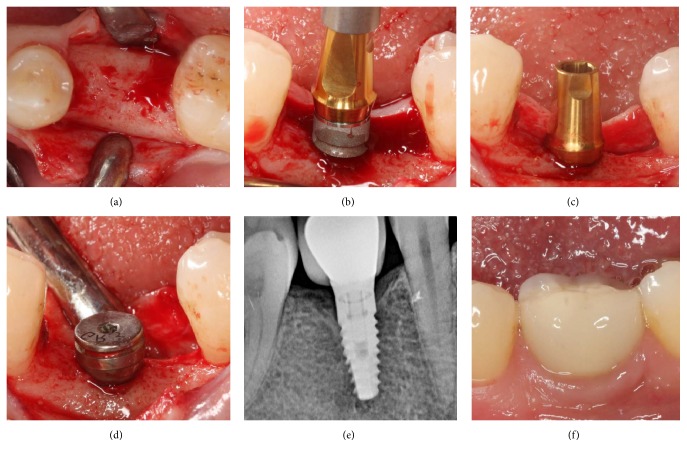
Full-thickness flaps were raised (a); M group implants were placed 0.3 mm supracrestally (b, c), loaded after a 3-month transmucosal healing period (d–f).

**Figure 3:**
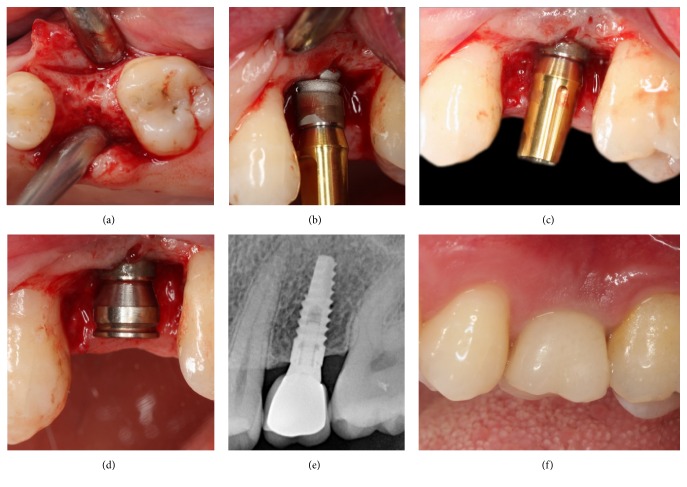
Full-thickness flaps were raised (a), L group implants were placed 1 mm supracrestally (b, c), loaded after a 3-month transmucosal healing period (d–f).

All surgical procedures were conducted under local anesthesia (Ultracain DS Forte, Sanofi Aventis, Istanbul, Turkey) by one clinician (BAG). In brief, a midcrestal incision was made, full-thickness flaps were raised, and implants were placed using the same surgical kit in both groups according to the manufacturer's instructions. The M group implants were placed 0.3 mm supracrestally, while the L group implants were placed 1 mm supracrestally (Figures 2 and 3). The mesiodistal aspect of the alveolar ridge was used as a reference point. Healing abutments were attached to the implants for transmucosal healing, and the mucoperiosteal flaps were sutured (Dogsan Medical Supplies Industry, Trabzon, Turkey). Postoperative medications included antibiotics (1000 mg amoxicillin and clavulanic acid twice daily for 7 days, starting on the day of surgery); an analgesic (600 mg ibuprofen as required every 6 hours); and 0.2% chlorhexidine mouthwash twice daily for 2 weeks, starting on the day after surgery. Sutures were removed 7 days after surgery. After a 3-month healing period, impressions were recorded with polyvinyl siloxane (Panasil, Kettenbach, Eschenburg, Germany) loaded on standard closed trays. Final laboratory-manufactured standard titanium abutments (Biohorizons) were attached to the implants and torqued to 30 N·cm as recommended by the manufacturer. Finally, cement-retained porcelain-fused-to-metal permanent restorations were cemented using zinc polycarboxylate cement (Adhesor Carbofine, Spofa Dental, Prague, Czech Republic).

### 2.4. Study Variables

The primary predictor variable was implant collar texture design. The primary outcome variable was marginal bone loss. Other study variables were implant dimensions, implant region, and probing depth.

### 2.5. Measurement of Marginal Bone Loss

All implant placement sites were radiographed with the long-cone paralleling technique using a digital radiography system (Kodak DS, Rochester, NY, USA) at baseline at 3 months and 1, 2, and 3 years after implant placement to evaluate vertical bone levels. The known implant length was used as a reference to increase measurement accuracy and eliminate the magnification factor in periapical radiographs. The distance between the mesial and distal edges of the implant shoulder and the most coronal level of the bone in contact with the implant body was calculated using a software program (Kodak DS). For each implant, the mean of the mesial and distal measurements was used. The baseline measurement was used as a reference point for comparisons. All measurements were evaluated by a single calibrated examiner (AS) specialized in periodontology to prevent bias and repeated with excellent reliability (*R* = 0.960).

### 2.6. Clinical Follow-Up for Evaluation of Peri-Implant Health Indices

All patients were recalled at 1 month after prosthesis delivery and at 12, 24, and 36 months after implant placement. The findings of clinical examinations were analyzed according to Albrektsson's success criteria [7]. All clinical parameters (probing depth [PD], modified plaque index [PI], and Mombelli's gingival index [GI]) were measured by a single periodontologist (AS) [8] at each follow-up visit. Peri-implant health indices were evaluated using a customized guide created after the final restorations were cemented to provide a reference point for probe measurements and reproducibility. Peri-implant PD was measured at the mesial, distal, midfacial, and palatal aspects of each implant using a periodontal probe (Periowise, Premier Dental Products Co., Plymouth Meeting, PA, USA), and the average was calculated for statistical evaluation. The GI was used to document soft tissue inflammation, while the PI was used to assess plaque accumulation. Oral hygiene instructions were also reinforced for patients if necessary. The annual prosthetic evaluation included a check of occlusion and articulation by a single prosthodontist (PG). Any adverse events associated with the prostheses, such as porcelain chipping, abutment screw loosening, decementation, and fracture, were recorded.

### 2.7. Statistical Analysis

Using G^*∗*^Power (version 3.1.9.2) and experience from a previous study [3], a minimum sample size of 74 implants was calculated to be necessary to detect a >0.2 ± 0.3 mm difference in bone loss between the M and L implants at the *α* = 0.05 level. An estimated dropout rate of 20% and a statistical power of 80% were also allowed for and an approximate total of 89 implants were deemed necessary for the study. The charts were reviewed and the data were collected by one researcher (PG). The data were entered into tables. Number Cruncher Statistical System 2007 software (NCSS, Kaysville, UT, USA) was used for the statistical analyses. The Shapiro-Wilk test was used to assess the normality of the data distribution. Because distribution of the data did not meet the requirements for normality and homogeneity of variance assumptions, nonparametric quantitative data were compared between groups using the Mann–Whitney *U* test and within groups using the Friedman test, and the Wilcoxon signed-rank test was used for pairwise comparisons. Three-way repeated measures ANOVA was used to assess the marginal bone loss variation related to other variables (region, length, and diameter). Linear regression analysis was conducted for mean, mesial, and distal marginal bone loss values, where group, region, diameter, and length were introduced as independent risk factors. The confidence interval was set to 95% and *p* < 0.05 was considered statistically significant.

## 3. Results and Discussion

### 3.1. Results

From the 68 patients selected for the study, 6 were excluded because marginal bone loss could not be measured accurately on their radiographs. Consequently, 62 patients (34 women and 28 men) of mean age 52.24 ± 13.38 (range 23–76) years who received 103 implants (56 in the M group; 47 in the L group) were included in our analysis (Table 1).

There was no significant difference between groups with regard to age, sex distribution, diameter, length, region, and restoration type (*p* > .05; Table 2).

All implants showed satisfactory clinical osseointegration, with a 3-year cumulative survival rate of 100% as assessed by Albrektsson's criteria.

Marginal bone loss was lower in the L group than in the M group at all time points (Mann–Whitney *U* test, *p* < 0.01; Table 3), being 0.49 (0.47, 0.5) mm in the L group (mesial 0.45; distal 0.52) and 1.38 (1.29, 1.46) mm in the M group (mesial 1.31; distal 1.45) at 3 years. However, marginal bone loss increased with time in both groups (Friedman test, *p* < 0.01), with the maximum observed at 1 year after implant placement. Marginal bone loss was also lower in the L group than in the M group for all subgroup comparisons (region, length, and diameter) (Table 4).

Three separate linear regression analyses were conducted for mean, mesial, and distal marginal bone loss values, where group, region, diameter, and length were introduced as independent risk factors (Table 5). All three models were found to be statistically significant with 0.578, 0.592, and 0.516 adjusted *R*2 levels (*F*: 35.975, *p*: 0.001, *f*: 37.935, *p*: 0.001, *F*: 28.148, and *p*: 0.001, resp.). Group and diameter factors had statistically significant effects on mean, mesial, and distal marginal bone loss values, where region and length factors did not have any contribution to the models. Being on the L group had similar effects on three marginal bone loss values, 0.411, 0.401, and 0.422, respectively, compared to M group. Having a diameter of 4.6 had similar effects on three marginal bone loss values, 0.241, 0.262, and 0.221, respectively, compared to 3.8 diameter group.

All patients showed satisfactory oral hygiene during the study (scores 0-1, data not shown), with no significant differences in GI and PI scores at any time point between the two groups.

An increase in PD was observed in both groups (*p* < 0.01; Mann–Whitney *U* Test; Table 2), however, a shallower PD was found for L group implants at all time points (*p* < 0.01; Friedman test; Table 2).

With regard to complications, porcelain fracture and screw loosening were observed at 2 years after loading in 3 patients, who consequently received new restorations. There were no other biomechanical complications.

### 3.2. Discussion

In this study, we compared marginal bone loss between subgingivally placed, short-collar posterior implants with machined collars (M group) and those with machined and laser-microtextured collars (L group) before and after loading. Currently, evidence supporting ideal implant collar configurations and length that limit marginal bone loss is lacking in the literature. The specific aims of this study were to compare marginal bone loss between two different short-collar implants that were used routinely and to evaluate clinical health on the basis of peri-implant tissue parameters.

The coronal portion of a two-piece dental implant system plays an important role in minimizing marginal bone loss [3, 9–11]. In systematic reviews investigating implant neck configurations, a great range of marginal bone loss (0.07–2.32 mm) was observed [2, 9–11]. The difference in marginal bone loss scores between studies can be attributed to several biological and biomechanical factors, such as biologic width, microgap, platform switching, loading pattern, soft tissue biotype, structure and length of the implant collar, characteristics of the implant surface, insertion depth, study design, and oral hygiene [9, 10]. Marginal bone loss at implants is a complex problem that is not completely understood and is a key factor in the long-term success of any implant design [2, 9, 11]. In the present study, marginal bone loss was lower in the L group than in the M group over a 3-year follow-up period; thus, part of the null hypothesis for this study was rejected. Nevertheless, according to Albrektsson's criteria, all implants were successful with regard to marginal bone loss [7]. The collars of the L group implants featured specific microchannels created by laser ablation to achieve a firm attachment with connective tissue [5], which can inhibit apical migration of epithelial tissue and decrease the detrimental effects of inflammatory cell infiltration caused by microgaps on marginal bone [12]. Another possible factor may be vertical differences in the location of the microgaps according to the marginal bone level in the two groups. Ericsson et al. [12] showed that inflammatory cell infiltration associated with microgaps extends 0.5–0.6 mm apically on all surfaces of a two-piece implant. Because of differences in collar configuration, microgaps were positioned 0.3 mm coronal to the crestal bone in the M group implants but 1 mm coronal to the crestal bone in the L group implants. Alomrani et al. found that implants with shorter machined coronal collars can prevent marginal bone loss [13]. However, our observations for the M group indicate that inflammatory cell infiltration may occur and cause resorption even if the implants have short machined collars with supracrestally positioned microgaps. In another study, Brägger et al. found 0.78 mm of marginal bone loss at 1 year after loading for supracrestally placed implants with the smooth collar placed above bone level [14]; this bone loss was greater than that in the L group in our study, even though the microgap was located far from the bone crest. These findings indicate that both specific collar surface characteristics and collar length above the bone can affect marginal bone loss. Iorio-Siciliano et al. compared laser-microtextured and machined collar implants according to marginal bone loss and soft tissue conditions as in our study, but the implants in both groups were placed with the smooth color portion at the level of the bone crest and healed in a two-stage protocol [15]. Although the collar level of the implants in the study by Iorio-Siciliano et al. was not comparable with that in the current study, their results with regard to marginal bone loss are consistent with our results; that is, the laser-microtextured collar may enable less bone resorption than implants with the smooth collar. The 1 mm supracrestal placement of implants with laser-microtextured collars may show limited bone resorption to a greater extent than the 0.3 mm supracrestal placement of implants with machined collars in a subgingival protocol. This protocol offers several benefits. First, resorption associated with microgap and micromovement (implant-abutment interface) may not be observed. Second, esthetic problems associated with high machined collar lengths can be minimized. Third, more benefit of a physical connective tissue attachment onto a laser-microtextured surface implant collar may be provided compared to when the implant is placed crestally or subcrestally. Fourth, the detrimental effect of residual cement to peri-implant health may be less for implants placed supracrestally in cases of cement-retained restorations [11, 13, 16]. Botos et al. [17] found marginal bone loss of 0.42 mm at 1 year after loading for implants with the same laser-microtextured collar design used in the present study, although these implants were placed crestally. In another study, Iorio-Siciliano et al. observed approximately 0.72 mm bone loss around platform-switched laser-microtextured collar implants that were placed 1 mm subcrestally after 2 years of follow-up [18]. Connective tissue attaches in a perpendicular manner to laser microgrooves on the implant collar and might prevent marginal bone loss [6, 12]. Placement of an implant laser-microtextured collar under the crestal bone may limit the ability of this special surface to prevent marginal bone loss when compared with supracrestal placement. In the study by Iorio-Siciliano et al., the platform-switched component of the collar may have been more effective in reducing crestal bone loss than a special laser-microtextured surface attachment, because the laser-microtextured surface of the implant was in contact mostly with bone and not with connective tissue. Considering these findings, it remains debatable whether the lesser bone resorption observed in the L group in our study was a result of the increased distance between microgaps and the marginal bone or of the firm attachment of connective tissue to the implant surface (biological seal). We speculate that both factors affect bone resorption synergistically.

The maximum bone loss was observed at 1 year after implant placement in both groups in the present study. This result is in accordance with those of several other clinical studies [9–11, 19]. The effects of adaptational factors such as surgical trauma, microgap-related bacterial contamination, host immune responses, loading forces, and biological width on marginal bone loss have been found to decrease after 1 year [3, 9–11]. These biological and mechanical changes may have influenced marginal bone loss immediately after implant insertion and continued for a year in both of our study groups.

Both the M group and L group implants showed increased marginal bone loss before loading. Koutouzis et al. [19] found marginal bone loss of 0.18 ± 0.27 mm at 4 months before loading. In another study, the mean marginal bone levels were 0.05 mm and 0.27 mm for two different implant designs at 1 year after loading [20]. Botos et al. [17] found marginal bone loss of 0.15 mm at 6 months before loading for implants with the same laser-microtextured collar design used in the present study. In our study, marginal bone loss was 0.68 mm and 0.28 mm for the M group and L group implants, respectively, before loading. One explanation for this increased marginal bone loss before loading was the removal and reconnection of healing abutments at least four times for implant stability measurements (data not shown) in our study. Repeated reconnection of abutments may compromise the mucosal seal and result in a more apical epithelial attachment [21], which can increase marginal bone loss. However, the L group implants appeared to be more resistant to repeated reconnection than the M group, exhibiting less bone resorption.

Values for all clinical parameters, including PI, GI, and PD, increased after prosthesis delivery despite regular oral hygiene appointments (Table 6). However, plaque control and soft tissue health were acceptable in both groups, resulting in an absence of peri-implantitis. PD values were significantly lower in the L group than in the M group, consistent with previous findings for implants with the same laser-microtextured collar design used in this study [16, 22]. This may be the result of increased resistance to probing as dense connective tissue attempts to establish a better mucosal barrier in the collar region of these implants [5]. Finally, the prosthetic outcomes were excellent in all cases, with screw loosening and porcelain chipping observed in only a small number of patients. Small adaptations and occlusal adjustments during recall visits may help to prevent such complications, although a 3-year observation period may be too short for mechanical problems to manifest [23].

This study has some limitations. First was the retrospective design, because patients and implant groups were not randomized, masked, or equally distributed with regard to the study variables. Subjects were evaluated according to a retrospective chart review after considering the study inclusion and exclusion criteria. Another limitation is the lack of evaluation of buccal and lingual marginal bone loss. Two-dimensional radiographs only show the mesial and distal aspects of an implant; however, stable buccal bone levels also affect the outcomes of implant-supported restorations [24]. Another limitation is the study design. All implants were placed in optimum conditions, with no grafted sites, minimally invasive flap elevation, and conventional loading protocols.

## 4. Conclusions

Within the limitations of this study, our results suggest predictable outcomes with regard to marginal bone loss and success for both implants with machined and laser-microtextured collars and those with machined collars; however, bone resorption is lower with the former than with the latter, before and after loading. The laser-microtextured collar may provide a shallower PD than machined collar implants. Additional long-term clinical investigations are necessary to clarify our findings further.

## Figures and Tables

**Table 1:** Descriptive summary of the study sample.

Study variable	Descriptive statistics
*Sample size, n (%)*	
Patients	62
Implants	103

*Demographic variables*	
Gender, M/F, *n (%)*	50 (48.5)/53 (51.5)
Age (years), *mean ± sd (min–max)*	52.24 ± 13.38 (23–76)

*Health status variables*	
ASA classification	
I	103 (%100)
Type of implant (*n* = 103), *n* (%)	
M	56 (54.4)
L	47 (45.6)
Region, *n* (%)	
Mandible (L/M)	30 (29.1)/35 (34.0)
Maxilla (L/M)	17 (16.5)/21 (20.4)
Diameter, *n* (%)	
3.8 (L/M)	35 (34.0)/42 (40.8)
4.6 (L/M)	12 (11.7)/14 (13.6)
Length, *n* (%)	
10.5 (L/M)	31 (30.1)/41 (39.8)
12 (L/M)	16 (15.5)/15 (14.6)
Restoration, *n* (%)	
SC (L/M)	23 (22.3)/22 (21.4)
FPD (L/M)	24 (23.3)/34 (33.0)

ASA, American Society of Anesthesiology; M, machined collar group; L, machined and laser-microtextured collar group; SC, single crown; FPD, fixed partial denture.

**Table 2:** Study variables versus predictor variable (M and L).

	Total (*n* = 103)	L (*n* = 47)	M (*n* = 56)	*p*
	Median (*Q*_1_, *Q*_3_)	Median (*Q*_1_, *Q*_3_)	Median (*Q*_1_, *Q*_3_)	
Age (years)	53 (46, 62)	49 (44, 62)	55.5 (48, 62.5)	^a^ **0.094**
Gender, F/M, *n*	53/50	23/24	30/26	^b^ **0.639**
Diameter, 3.8/4.6, *n*	77/26	35/12	42/14	^b^ **0.951**
Length, 10.5/12, *n*	72/31	31/16	41/15	^b^ **0.434**
Region, max/mand, *n*	38/65	17/30	21/35	^b^ **0.889**
Restoration, SC/FPD, *n*	45/58	23/24	22/34	^b^ **0.325**

^a^Mann–Whitney *U* test;  ^b^Pearson chi-square test.

M, machined collar group; L, machined and laser-microtextured collar group; SC, single crown; FPD, fixed partial denture.

**Table 3:** Marginal bone loss with M group and L group implants at the 3-year follow-up examination.

	L (*n* = 47)						M (*n* = 56)						^a^ *p*_mean_	^a^ *p*_mesial_	^a^ *p*_distal_
	Mean		Mesial		Distal		Mean		Mesial		Distal				
	Median (*Q*_1_, *Q*_3_)		Median (*Q*_1_, *Q*_3_)		Median (*Q*_1_, *Q*_3_)		Median (*Q*_1_, *Q*_3_)		Median (*Q*_1_, *Q*_3_)		Median (*Q*_1_, *Q*_3_)				
3 m	0.27 (0.25, 0.29)		0.23 (0.22, 0.28)		0.29 (0.25, 0.31)		0.68 (0.65, 0.72)		0.65 (0.61, 0.69)		0.71 (0.69, 0.74)		**0.001** ^*∗∗*^	**0.001** ^*∗∗*^	**0.001** ^*∗∗*^
12 m	0.47 (0.43, 0.48)		0.41 (0.39, 0.46)		0.50 (0.46, 0.54)		1.33 (1.25, 1.42)		1.26 (1.08, 1.35)		1.41 (1.27, 1.47)		**0.001** ^*∗∗*^	**0.001** ^*∗∗*^	**0.001** ^*∗∗*^
24 m	0.48 (0.45, 0.5)		0.43 (0.40, 0.46)		0.51 (0.47, 0.55)		1.37 (1.28, 1.45)		1.30 (1.18, 1.40)		1.43 (1.31, 1.51)		**0.001** ^*∗∗*^	**0.001** ^*∗∗*^	**0.001** ^*∗∗*^
36 m	0.49 (0.47, 0.5)		0.45 (0.42, 0.47)		0.52 (0.48, 0.55)		1.38 (1.29, 1.46)		1.31 (1.20, 1.41)		1.45 (1.32, 1.53)		**0.001** ^*∗∗*^	**0.001** ^*∗∗*^	**0.001** ^*∗∗*^

	^c^ **0.001** ^*∗∗*^		^c^ **0.001** ^*∗∗*^		^c^ **0.001** ^*∗∗*^		^c^ **0.001** ^*∗∗*^		^c^ **0.001** ^*∗∗*^		^c^ **0.001** ^*∗∗*^				

	Median (*Q*_1_, *Q*_3_)	^b^ *p*	Median (*Q*_1_, *Q*_3_)	^b^ *p*	Median (*Q*_1_, *Q*_3_)	^b^ *p*	Median (*Q*_1_, *Q*_3_)	^b^ *p*	Median (*Q*_1_, *Q*_3_)	^b^ *p*	Median (*Q*_1_, *Q*_3_)	^b^ *p*	^a^ *p*	^a^ *p*	^a^ *p*

3 m–12 m	0.20 (0.16, 0.23)	**0.001** ^*∗∗*^	0.18 (0.13, 0.21)	**0.001** ^*∗∗*^	0.22 (0.17, 0.25)	**0.001** ^*∗∗*^	0.65 (0.54, 0.71)	**0.001** ^*∗∗*^	0.63 (0.41, 0.70)	**0.001** ^*∗∗*^	0.69 (0.56, 0.76)	**0.001** ^*∗∗*^	**0.001** ^*∗∗*^	**0.001** ^*∗∗*^	**0.001** ^*∗∗*^
3 m–24 m	0.22 (0.17, 0.24)	**0.001** ^*∗∗*^	0.20 (0.15, 0.23)	**0.001** ^*∗∗*^	0.23 (0.18, 0.26)	**0.001** ^*∗∗*^	0.69 (0.59, 0.74)	**0.001** ^*∗∗*^	0.67 (0.49, 0.74)	**0.001** ^*∗∗*^	0.70 (0.58, 0.80)	**0.001** ^*∗∗*^	**0.001** ^*∗∗*^	**0.001** ^*∗∗*^	**0.001** ^*∗∗*^
3 m–36 m	0.23 (0.18, 0.25)	**0.001** ^*∗∗*^	0.20 (0.17, 0.23)	**0.001** ^*∗∗*^	0.24 (0.18, 0.27)	**0.001** ^*∗∗*^	0.70 (0.63, 0.77)	**0.001** ^*∗∗*^	0.70 (0.53, 0.77)	**0.001** ^*∗∗*^	0.72 (0.59, 0.83)	**0.001** ^*∗∗*^	**0.001** ^*∗∗*^	**0.001** ^*∗∗*^	**0.001** ^*∗∗*^
12 m–24 m	0.02 (0.01, 0.02)	**0.001** ^*∗∗*^	0.02 (0.01, 0.03)	**0.001** ^*∗∗*^	0.01 (0, 0.02)	**0.001** ^*∗∗*^	0.03 (0.02, 0.04)	**0.001** ^*∗∗*^	0.03 (0.01, 0.05)	**0.001** ^*∗∗*^	0.02 (0.01, 0.04)	**0.001** ^*∗∗*^	**0.001** ^*∗∗*^	**0.013** ^*∗*^	**0.001** ^*∗∗*^
12 m–36 m	0.02 (0.02, 0.03)	**0.001** ^*∗∗*^	0.03 (0.01, 0.04)	**0.001** ^*∗∗*^	0.02 (0.01, 0.02)	**0.001** ^*∗∗*^	0.04 (0.03, 0.05)	**0.001** ^*∗∗*^	0.04 (0.03, 0.07)	**0.001** ^*∗∗*^	0.04 (0.02, 0.05)	**0.001** ^*∗∗*^	**0.001** ^*∗∗*^	**0.001** ^*∗∗*^	**0.001** ^*∗∗*^
24 m–36 m	0.01 (0.01, 0.02)	**0.001** ^*∗∗*^	0.01 (0, 0.02)	**0.001** ^*∗∗*^	0.01 (0, 0.01)	**0.001** ^*∗∗*^	0.01 (0.01, 0.02)	**0.001** ^*∗∗*^	0.02 (0.01, 0.02)	**0.001** ^*∗∗*^	0.01 (0, 0.02)	**0.001** ^*∗∗*^	**0.001** ^*∗∗*^	**0.007** ^*∗∗*^	**0.017** ^*∗*^

^a^Mann–Whitney *U* test, ^b^Wilcoxon signed ranks test, and ^c^Friedman test.

^*∗*^ *p* < 0.05; ^*∗∗*^ *p* < 0.01.

**Table 4:** Three-way repeated measures ANOVA results for mesial, distal, and mean marginal bone loss related to other variables (region, length, and diameter).

	Within-subjects effects			Between-subjects effects		
	Source	^‡^ *F*	*p*	Source	*F*	*p*
Mean	Time	392.271	0.001^*∗∗*^	Intercept	1613.261	0.001^*∗∗*^
	Time *∗* Group	77.335	0.001^*∗∗*^	Group	298.065	0.001^*∗∗*^
	Time *∗* Region	0.382	0.577	Region	0.547	0.461
	Time *∗* Group *∗* Region	0.410	0.562	Group *∗* Region	0.220	0.640
	Time	322.461	0.001^*∗∗*^	Intercept	1533.668	0.001^*∗∗*^
	Time *∗* Group	67.721	0.001^*∗∗*^	Group	287.963	0.001^*∗∗*^
	Time *∗* Diameter	28.501	0.001^*∗∗*^	Diameter	26.997	0.001^*∗∗*^
	Time *∗* Group *∗* Diameter	5.465	0.014^*∗*^	Group *∗* Diameter	5.561	0.019^*∗*^
	Time	365.332	0.001^*∗∗*^	Intercept	1477.272	0.001^*∗∗*^
	Time *∗* Group	76.285	0.001^*∗∗*^	Group	276.488	0.001^*∗∗*^
	Time *∗* Length	1.040	0.324	Length	0.875	0.352
	Time *∗* Group *∗* Length	0.101	0.799	Group *∗* Length	0.001	0.972

Mesial	Time	333.461	0.001^*∗∗*^	Intercept	1626.138	0.001^*∗∗*^
	Time *∗* Group	69.415	0.001^*∗∗*^	Group	319.692	0.001^*∗∗*^
	Time *∗* Region	0.417	0.566	Region	0.787	0.377
	Time *∗* Group *∗* Region	0.686	0.442	Group *∗* Region	0.634	0.428
	Time	277.718	0.001^*∗∗*^	Intercept	1550.248	0.001^*∗∗*^
	Time *∗* Group	60.532	0.001^*∗∗*^	Group	312.604	0.001^*∗∗*^
	Time *∗* Diameter	33.225	0.001^*∗∗*^	Diameter	27.627	0.001^*∗∗*^
	Time *∗* Group *∗* Diameter	8.381	0.002^*∗∗*^	Group *∗* Diameter	6.420	0.013^*∗*^
	Time	307.962	0.001^*∗∗*^	Intercept	1479.121	0.001^*∗∗*^
	Time *∗* Group	70.307	0.001^*∗∗*^	Group	300.815	0.001^*∗∗*^
	Time *∗* Length	0.823	0.391	Length	0.872	0.353
	Time *∗* Group *∗* Length	0.246	0.676	Group *∗* Length	0.041	0.840

Distal	Time	372.087	0.001^*∗∗*^	Intercept	1460.545	0.001^*∗∗*^
	Time *∗* Group	69.780	0.001^*∗∗*^	Group	254.932	0.001^*∗∗*^
	Time *∗* Region	0.285	0.640	Region	0.338	0.563
	Time *∗* Group *∗* Region	0.160	0.740	Group *∗* Region	0.030	0.864
	Time	286.418	0.001^*∗∗*^	Intercept	1342.527	0.001^*∗∗*^
	Time *∗* Group	58.223	0.001^*∗∗*^	Group	236.093	0.001^*∗∗*^
	Time *∗* Diameter	18.651	0.001^*∗∗*^	Diameter	23.370	0.001^*∗∗*^
	Time *∗* Group *∗* Diameter	2.458	0.112	Group *∗* Diameter	4.434	0.038^*∗*^
	Time	349.141	0.001^*∗∗*^	Intercept	1343.308	0.001^*∗∗*^
	Time *∗* Group	67.089	0.001^*∗∗*^	Group	232.828	0.001^*∗∗*^
	Time *∗* Length	1.103	0.309	Length	0.798	0.374
	Time *∗* Group *∗* Length	0.022	0.920	Group *∗* Length	0.055	0.815

^‡^Assumption of sphericity was violated; Greenhouse-Geisser *F* and *p* values are reported.

^*∗*^ *p* < 0.05; ^*∗∗*^ *p* < 0.01.

**Table 5:** Linear regression analysis for mesial, distal, and mean marginal bone loss values.

	Source	Beta	*p*	95% CI for beta	
				Lower bound	Upper bound
Mean	Group (L)	0.411	0.001^*∗∗*^	0.334	0.488
	Region (mand)	0.008	0.852	−0.073	0.089
	Diameter (4.6)	0.241	0.001^*∗∗*^	0.152	0.331
	Length (12)	−0.010	0.824	−0.096	0.077

Mesial	Group (L)	0.401	0.001^*∗∗*^	0.326	0.476
	Region (mand)	0.011	0.781	−0.068	0.090
	Diameter (4.6)	0.262	0.001^*∗∗*^	0.175	0.349
	Length (12)	0.001	0.973	−0.083	0.086

Distal	Group (L)	0.422	0.001^*∗∗*^	0.334	0.509
	Region (mand)	0.004	0.927	−0.088	0.096
	Diameter (4.6)	0.221	0.001^*∗∗*^	0.119	0.323
	Length (12)	−0.021	0.673	−0.119	0.077

Dependent variables: difference between 3-month and 36-month mean, mesial and distal marginal bone loss values.

^*∗∗*^ *p* < 0.01.

**Table 6:** Comparison of probing depth parameters after prosthesis delivery between the L group and M group implants.

Months	L (*n* = 47)		M (*n* = 56)		^a^ *p*
	Median (*Q*_1_, *Q*_3_)		Median (*Q*_1_, *Q*_3_)		
4	1.75 (1.5, 1.75)		2.5 (2.5, 2.75)		0.001^*∗∗*^
12	2 (1.75, 2)		3 (3, 3.25)		0.001^*∗∗*^
24	2 (2, 2.25)		3.25 (3.25, 3.5)		0.001^*∗∗*^
36	2.25 (2, 2.5)		3.5 (3.25, 3.5)		0.001^*∗∗*^

	^d^0.001^*∗∗*^		^d^0.001^*∗∗*^		

	Median (*Q*_1_, *Q*_3_)	^c^ *p*	Median (*Q*_1_, *Q*_3_)	^c^ *p*	^a^ *p*

4–12	0.25 (0.25, 0.25)	0.001^*∗∗*^	0.5 (0.25, 0.5)	0.001^*∗∗*^	0.001^*∗∗*^
4–24	0.5 (0.25, 0.5)	0.001^*∗∗*^	0.75 (0.5, 0.75)	0.001^*∗∗*^	0.001^*∗∗*^
4–36	0.5 (0.5, 0.75)	0.001^*∗∗*^	0.75 (0.5, 1)	0.001^*∗∗*^	0.001^*∗∗*^
12–24	0.25 (0, 0.25)	0.001^*∗∗*^	0.25 (0.25, 0.25)	0.001^*∗∗*^	0.463
12–36	0.25 (0.25, 0.5)	0.001^*∗∗*^	0.25 (0.25, 0.5)	0.001^*∗∗*^	0.507
24–36	0.25 (0, 0.25)	0.001^*∗∗*^	0 (0, 0.25)	0.001^*∗∗*^	0.239

^a^Mann–Whitney *U* test; ^c^Wilcoxon signed-rank test; ^d^Friedman test. ^*∗∗*^ *p* < 0.01. *Q*_1_: first quartile, 25th percentile; *Q*_3_: third quartile, 75th percentile.
